# LRP8 Regulates Lipid Metabolism to Stimulate Malignant Progression and Cisplatin Resistance in Bladder Cancer

**DOI:** 10.1002/kjm2.70042

**Published:** 2025-05-15

**Authors:** Gang‐Feng Wu, Zhen‐Gang Luo, Ke Gao, Yu Ren, Chong Shen, Xiang‐Rong Ying

**Affiliations:** ^1^ Department of Urology Shaoxing People's Hospital Shaoxing China

**Keywords:** bladder cancer, cisplatin resistance, lipid metabolism, LRP8

## Abstract

Low‐density lipoprotein receptor‐related protein 8 (LRP8) is a crucial regulator of lipid metabolism and is implicated in the development and treatment of various cancers. However, its role in bladder cancer (BCa) remains unknown. We analyzed LRP8 expression in BCa using the TCGA database and clinical samples. We manipulated LRP8 expression in tumor cell lines using siRNA or overexpression plasmid transfection. Cell proliferation, migration, invasion, apoptosis, and drug resistance were assessed through CCK‐8, transwell, flow cytometry, and IC_50_ assays. Additionally, a rescue experiment confirmed the association between LRP8 and lipid metabolism. LRP8 was significantly upregulated in BCa tissues and cells. Knockdown of LRP8 reduced tumor cell proliferation, migration, invasion, and increased apoptosis while enhancing cisplatin sensitivity. Overexpression of LRP8 boosted malignant progression and cisplatin resistance in tumor cells. The expression level of LRP8 is positively linked with the expression of lipid metabolism‐related genes, phospholipid accumulation, and triglyceride accumulation. Notably, inhibiting lipid metabolism reversed the malignant progression and cisplatin resistance induced by LRP8 overexpression. LRP8 could promote BCa malignant progression and cisplatin resistance through lipid metabolism regulation.

## 1 Introduction

Bladder cancer (BCa) is one of the most prevalent global cancers, impairing the function of the urinary system. According to the reports, over 570,000 new BCa cases occur and more than 210,000 people die from BCa every year, which imposes a heavy burden on economic development [1]. BCa is regularly classified into nonmuscle‐invasive BCa, muscle‐invasive BCa, and metastatic BCa based on the variations in their presentations. Among them, muscle‐invasive BCa and metastatic BCa are often extremely difficult to treat and thus have a high mortality rate, with a 5‐year survival rate of less than 70% [2]. To deal with this situation, patients are given various treatments including surgery, radiotherapy, chemotherapy, bacillus Calmette‐Guerin (BCG) infusion, and immunotherapy. Among these, chemotherapy is widely used due to its stable effectiveness, and for perioperative and first‐line metastatic settings, cisplatin‐based chemotherapy is still the standard treatment [3]. Unfortunately, the efficacy of cisplatin has been greatly reduced by drug resistance. Based on relevant statistics, only 50% of patients respond to platinum treatment, and a large proportion of patients experience cancer progression or tumor recurrence during the treatment [4]. Therefore, it is anticipated that addressing the issue of cisplatin resistance in BCa will greatly elevate patient survival.

Lipid metabolism is a mechanism by which tumor cells can obtain the necessary materials and energy for proliferation and metastasis. Dysregulated lipid metabolism is one of the most prominent metabolic alterations in cancer [5]. Relevant research has proved that this alteration not only regulates tumor development but also affects the chemoresistance of tumor cells [6]. In the study of Liu et al. [7], lncBBET can regulate the lipid metabolism of BCa cells by activating the PPAR signaling pathway to stimulate the BCa malignant progression in vivo and in vitro. Liu et al. [8] discovered that the long noncoding RNA, lncROPM, can activate downstream signaling by regulating phospholipid metabolism and the free fatty acids (FAs) production, thereby promoting the stemness maintenance of breast stem cells and ultimately affecting the chemoresistance of breast cancer (BC) cells. Existing research indicates a strong correlation between dysregulated lipid metabolism and cisplatin resistance in BCa cells [9]. Taken together, lipid metabolism is essential in the BCa malignant development and cisplatin resistance; therefore, the treatment of BCa is expected to benefit from the lipid metabolism pathway regulation. However, the relevant research in BCa is not in‐depth, lacking the investigation of safe and efficient regulatory targets [10].

Low‐density lipoprotein receptor‐related protein 8 (LRP8) has been widely studied in cancer, and related research has evidenced that highly expressed LRP8 can facilitate the development of lung cancer (LC), BC, prostate cancer, gastric cancer (GC), and melanoma [11]. However, the function of LRP8 in BCa remains unclear. It is worth noting that LRP8 has been demonstrated as a key gene involved in regulating lipid metabolism [12]. Additionally, lipid metabolism reprogramming is a crucial mechanism mediating chemotherapy resistance in BCa [9]. Therefore, it is highly likely that LRP8 may influence the chemotherapy resistance level in BCa by modulating lipid metabolism. This speculation has already been reflected in studies on hepatocellular carcinoma (HCC) and LC. For example, studies by Cai et al. [13] have indicated that the β‐catenin expression can be activated by LRP8 to further suppress the apoptosis and chemotherapy sensitivity of HCC cells. Qiu et al. [14] verified that the cisplatin sensitivity of LC cells can be inhibited by LRP8 overexpression. However, whether LRP8 plays a similar role in BCa is still unknown.

To address this issue, the LRP8 expression in BCa was examined and verified in this study. Then, through the regulation of LRP8 expression, investigations were conducted for the function of LRP8 in the malignant development and cisplatin resistance of BCa cells. Finally, the regulatory role of LRP8 in lipid metabolism in BCa cells was confirmed, and the mechanism by which LRP8 affected the malignant progression and cisplatin resistance of BCa cells was verified by interfering with lipid metabolism. These findings provide the first evidence of the role of LRP8 in the development and treatment of BCa while highlighting the impact of lipid metabolism reprogramming. The discovery of this regulatory mechanism offers potential therapeutic targets for addressing chemotherapy resistance in BCa.

## 2 Materials and Methods

### 2.1 Bioinformatics Analysis

The LRP8‐BCa expression dataset (19 normal tissues and 412 tumor tissues) was downloaded from The Cancer Genome Atlas (TCGA) database (https://portal.gdc.cancer.gov/) (access date: December 8, 2023). Next, the standardized expression profile data was extracted to conduct the differential analysis of LRP8 expression between the normal and tumor groups, and violin plots were graphed. Gene set enrichment analysis (GSEA v4.3.3) was performed to determine the enriched signaling pathways of LRP8 in BCa, and the signaling pathways related to lipid metabolism were screened. Pearson correlation analysis was conducted to validate the regulatory impact of LRP8 on lipid metabolism using selected lipid metabolism‐related genes.

### 2.2 Clinical Samples

In this study, the 10 tumor tissues and corresponding adjacent cancer tissues were obtained from BCa patients at Shaoxing People's Hospital from January 2023 to July 2023. All patients had given written informed consent. This study was conducted in accordance with the Declaration of Helsinki and approved by the Shaoxing People's Hospital Ethics Committee.

### 2.3 Cell Lines and Cultivation Conditions

BCa cells (T24, RT4, 5637) and the immortalized human uroepithelial cell line (SV‐HUC‐1) were acquired from Procell Life Science & Technology (China). SV‐HUC‐1 cells were cultivated in Ham's F‐12 K medium, the 5637 cells were in the RPMI‐1640 medium, and the T24 cells were in McCoy's 5A medium. 10% fetal bovine serum (FBS) and 1% penicillin/streptomycin were added to each medium. All cells were cultivated in an incubator at 37°C with 5% CO_2_.

### 2.4 Cell Transfection

To construct the oe‐LRP8 plasmid, the LRP8 coding sequence was synthesized and cloned into the pcDNA3.1 vector by GenePharma (China). The empty pcDNA3.1 plasmid was utilized as the negative control (oe‐NC). si‐LRP8 and si‐NC were designed and synthesized by RiboBio (China). Trypsin was used to digest cells that were well‐growing and in the logarithmic growth phase to obtain a cell suspension. Subsequently, in a six‐well plate, cells were seeded at a density of 1 × 10^6^ cells/well and cultivated until the confluence reached 70%. Transfection was conducted utilizing Lipofectamine LTX reagent and PLUS reagent (Thermo Fisher Scientific, USA). In brief, the cultivation medium was replaced with Opti‐MEM, and then 100 μL of Opti‐MEM was taken and mixed with PLUS reagent and plasmid or LTX reagent, respectively. Before being added to the medium, the plasmid solution was added to the LTX solution and incubated for 5 min. After 6 h, the medium was replaced with a complete culture medium. Transfection lasted for 48 h, and cells were collected for subsequent assays.

### 2.5 Immunohistochemistry (IHC) Assay

Following fixing with 4% paraformaldehyde, tissue samples were dehydrated and embedded in paraffin. The samples were divided into 4 μm sections, treated with gradient ethanol solutions after the removal of wax, and then treated with sodium citrate to repair the antigens. The sections were incubated overnight with primary antibody ApoER2/LRP8 Rabbit mAb (ABclonal, China) at 4°C after being blocked with 5% skim milk. Then the secondary antibody horseradish peroxidase (HRP)‐labeled Goat Anti‐Rabbit Immunoglobulin G (IgG) (H + L) (ABclonal, China) was added and incubated for 1 h at room temperature. The sections were then stained with hematoxylin and 3,3′‐diaminobenzidine tetrahydrochloride.

### 2.6 Quantitative Reverse Transcription Polymerase Chain Reaction (qRT‐PCR)

The total RNA from each group was extracted using RNAiso Plus (TAKARA, Japan) in accordance with the manufacturer's instructions. A NanoDrop ND‐1000 spectrophotometer (Thermo Fisher Scientific, USA) was utilized to measure the concentration and purity of RNA. Afterward, following the PrimeScript RT reagent kit (TAKARA, Japan) instructions, cDNA was generated by reverse transcription. Next, qRT‐PCR was carried out utilizing TB Green Premix Ex Taq II (TAKARA, Japan) on an Applied Biosystems 7500 fast real‐time fluorescence quantitative PCR system (Thermo Fisher Scientific, USA). The 2^−ΔΔCt^ method was utilized to determine the relative expression level of the target gene mRNA, with GAPDH serving as the reference gene. The primer sequences were bought from Tsingke Biotech (China). The primer sequences are presented in Table 1.

**TABLE 1:** Primer sequences.

Name	Primer sequence (5′ → 3′)
LRP8	F: GACGATGACTGCTTAGACCAC R: TCACACTTCCACCGTTCG
SREBF1	F: GCTCCCTAGGAAGGGCCGTA R: CGCCGACTTCACCTTCGAT
FASN	F: GCAAGCTGAAGGACCTGTCT R: AATCTGGGTTGATGCCTCCG
ACC1	F: ATCTTGAGGGCTAGGTCTTTTT R: AGAGTGCTGGTTCAGCTCC
β‐Actin	F: GCCGCCAGCTCACCAT R: TCGTCGCCCACATAGGAATC

### 2.7 Western Blot (WB)

Cells were lysed using radioimmunoprecipitation assay (RIPA) lysis buffer (Beyotime, China) containing protease inhibitors and phosphatase inhibitors, and then put on ice for 10 min. Centrifugation was performed for 10 min at 4°C and 12,000 rpm, and the supernatant was collected. The protein concentration was determined using the bicinchoninic acid (BCA) protein assay kit (Beyotime, China). The protein sample containing 1× sodium dodecyl sulfate‐polyacrylamide gel electrophoresis (SDS‐PAGE) protein loading buffer was denatured for 7 min at 100°C and then subjected to electrophoresis using 10% SDS‐PAGE. After that, the protein was moved to the PVDF membrane, and the membrane was blocked for 1 h with 5% skim milk. Subsequently, the samples were incubated at 4°C overnight with primary antibodies ApoER2/LRP8 Rabbit mAb, SREBP1 Rabbit pAb, FASN Rabbit pAb, or ACC1 Rabbit pAb (ABclonal, China). After washing with tris‐buffered saline tween‐20 (TBST), the samples were incubated with secondary antibody HRP‐labeled Goat Anti‐Rabbit IgG (H + L) (ABclonal, China) at room temperature for 1 h. Following one more washing with TBST, the Omni‐ECL ultra‐sensitive chemiluminescence kit (Epizyme, China) was used to develop color and capture band images through the ChemiScope 6000 chemiluminescence imaging system (Clinx, China).

### 2.8 Cell Counting Kit‐8 (CCK‐8) Assay for Cell Proliferation

Cell proliferation was assessed following the instructions of the CCK‐8 assay kit (Solarbio, China). Briefly, cell suspensions from each group were collected and a 96‐well plate was seeded with cells at a density of 2 × 10^3^ cells/well. After the cells adhered, 10 μL of CCK‐8 solution was added at 0, 24, 48, and 72 h, and cells were incubated for 2 h at 37°C. Absorbance was determined at 450 nm using the microplate reader assay kit.

### 2.9 Colony Formation Assay

Cell suspensions from each group were collected and cells were seeded at a density of 500 cells/well into a 12‐well plate. Cells were cultivated in fresh medium for 14 days, fixed with 4% paraformaldehyde, and stained with 0.1% crystal violet solution (Solarbio, China). The cells were washed several times with phosphate‐buffered saline (PBS), dried, and photographed with a digital camera.

### 2.10 Transwell Assay

The Transwell upper chamber (Corning, USA) was placed in a 24‐well plate. A serum‐free medium was used to dilute the Matrigel matrix (BD, USA) at a ratio of 1:8. 50 μL of the dilution was added to the Transwell upper chamber (without Matrigel coating during the migration experiment) and incubated for 2 h at 37°C with 5% CO_2_ to set the matrix gel. Cells from each group were collected and resuspended in a serum‐free medium. Next, 200 μL of cell suspension (2 × 10^4^ cells/well), while the lower chamber was replete with 600 μL of medium containing 10% FBS. The cells were incubated for 24 h, and then they were fixed with 4% paraformaldehyde and stained with 0.1% crystal violet solution (Solarbio, China). Under a microscope, cells were counted and photographed.

### 2.11 Flow Cytometry

To analyze the cell apoptosis rate, Annexin V‐FITC/PI Apoptosis Kit (MULTISCIENCES, China) was utilized for experiments strictly following the instructions. In short, the collected cells were centrifuged at 1000 rpm for 5 min, and the supernatant was discarded. Then, collected cells were washed once using PBS and carefully resuspended for counting. Next, 1 × 10^6^ cells were resuspended in 500 μL 1× Binding Buffer solution, and 5 μL Annexin V‐FITC and 10 μL propidium iodide (PI) were added in sequence. After gentle vortex mixing, cells were incubated in the dark at room temperature for 5 min, and then the carrying status of apoptosis markers in cells in each group was analyzed on a NovoCyte flow cytometer system (Agilent, USA).

### 2.12 CCK‐8 Assay for Half Maximal Inhibitory Concentration (IC_50_ )

5 × 10^3^ cells were seeded into a 96‐well plate. The supernatant was discarded once the cells had adhered. After adding a culture medium containing different concentrations of cisplatin to the wells, they were incubated at 37°C with 5% CO_2_ for 48 h. Then, after incubation with 10 μL CCK‐8 solution (Solarbio, China) for 2 h, a microplate reader was utilized to examine the optical density (OD) value at 450 nm, and the IC_50_ value was calculated using GraphPad Prism 8.

### 2.13 Lipid Content Measurement

Lipids were extracted from cell homogenate using chloroform/methanol (2:1) according to the instructions of the EnzyChrom phospholipids assay kit (BioAssay Systems, USA) and EnzyChrom triglycerides assay kit (BioAssay Systems, USA) to measure the lipid content of each component. In brief, standard samples were diluted in a gradient and stained with a dye solution, and the standard curve was plotted based on the OD value at 570 nm. Subsequently, the OD values of each group were examined and brought into the formula to calculate the corresponding lipid concentration.

Lipid metabolism inhibitor, Danthron was purchased from MedChemExpress (USA) [15].

### 2.14 Statistical Analysis

All data were presented as mean ± standard deviation (*n* = 3). Relevant results were analyzed and plotted using GraphPad Prism 8.3.0 software. One‐way analysis of variance (ANOVA) or the Student's *T*‐test was used to analyze significant differences (*indicates *p* < 0.05, with statistical significance; ns means no statistical significance).

## 3 Results

### 3.1 LRP8 Is Upregulated in BCa and Is Related to a Dismal Prognosis

**FIGURE 1:**
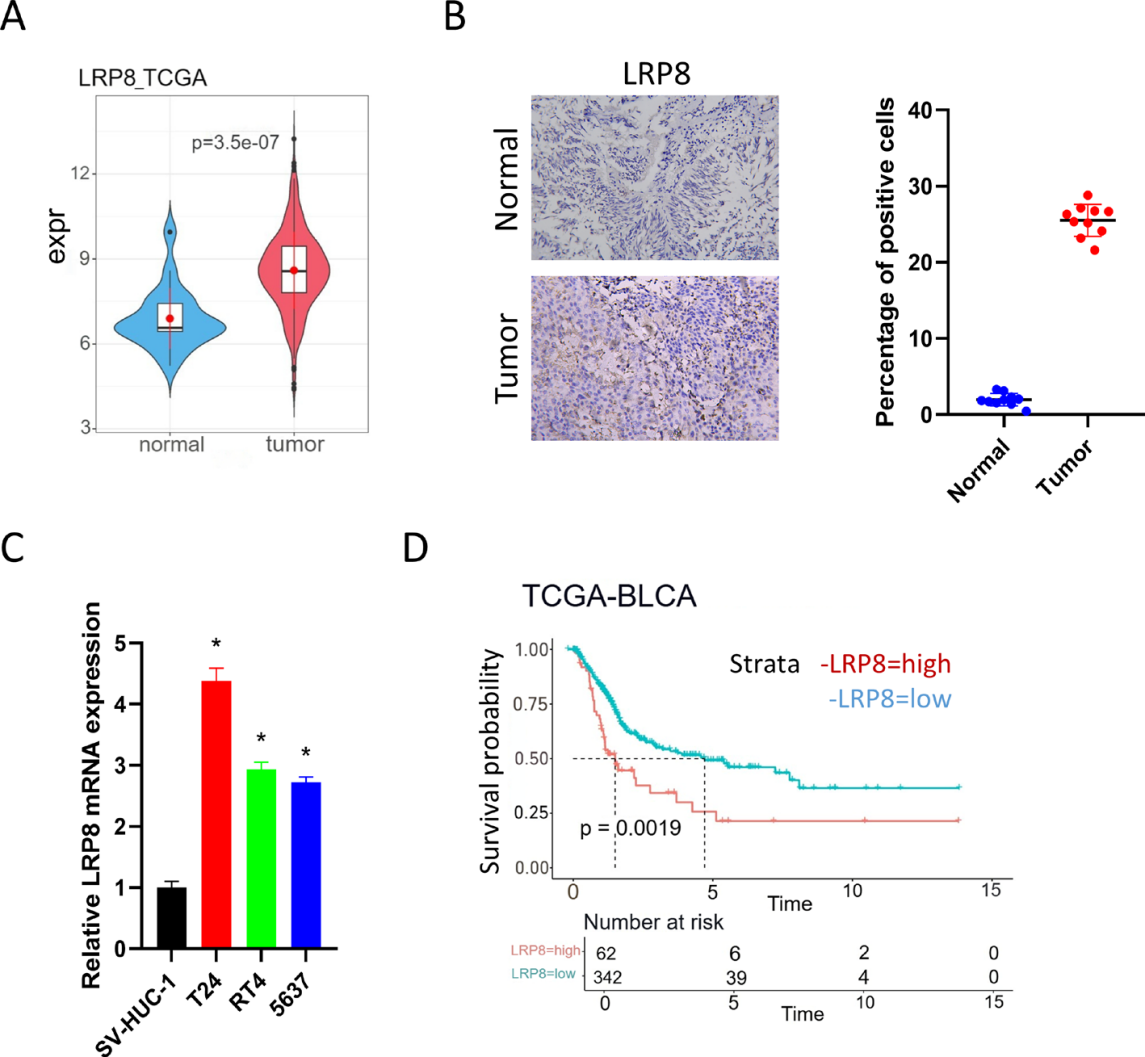
LRP8 is upregulated in BCa and is related to a dismal prognosis. (A) TCGA database analysis of the LRP8 expression in tumor tissues and adjacent tissues. (B) IHC detection of the LRP8 expression in tumor tissues and adjacent tissues (*n* = 10). The left picture is the representative image. (C) QRT‐PCR detection of the LRP8 expression in immortalized human uroepithelial cells and BCa cells. (D) The Kaplan–Meier survival curve was used to analyze the overall survival of patients with high or low LRP8 expression. * indicates *p* < 0.05, which means a significant difference. All experiments were repeated three times. BCa, bladder cancer; IHC, Immunohistochemistry; LRP8, low‐density lipoprotein receptor‐related protein 8; qRT‐PCR, Quantitative reverse transcription polymerase chain reaction; TCGA, The Cancer Genome Atlas Program.

To investigate the expression of LRP8 in BCa, this study collected and analyzed data from the TCGA database and performed validation in both tissue samples and cell lines. The TCGA results demonstrated that tumor tissues had a noticeable overexpression of LRP8 (Figure 1A). Next, the IHC assays were performed to determine the expression of LRP8 in tumor tissues and corresponding adjacent tissues. The results were consistent with the findings of bioinformatics analysis (Figure 1B). Furthermore, the expression of LRP8 in human urothelial immortalized cells (SV‐HUC‐1) and BCa cells (T24, RT4, 5637) were compared. According to qRT‐PCR results, the expression levels of LRP8 in all BCa cells were significantly higher than in the control group (Figure 1C). Furthermore, based on the clinical information collected from the TCGA database, compared with the LRP8 low‐expression group, the LPP8 high‐expression group had a significantly reduced overall survival prognosis (Figure 1D). Taken together, LRP8 is highly expressed in BCa and is associated with poor prognosis in patients.

### 3.2 LRP8 Promotes BCa Malignant Progression and Cisplatin Resistance

**FIGURE 2:**
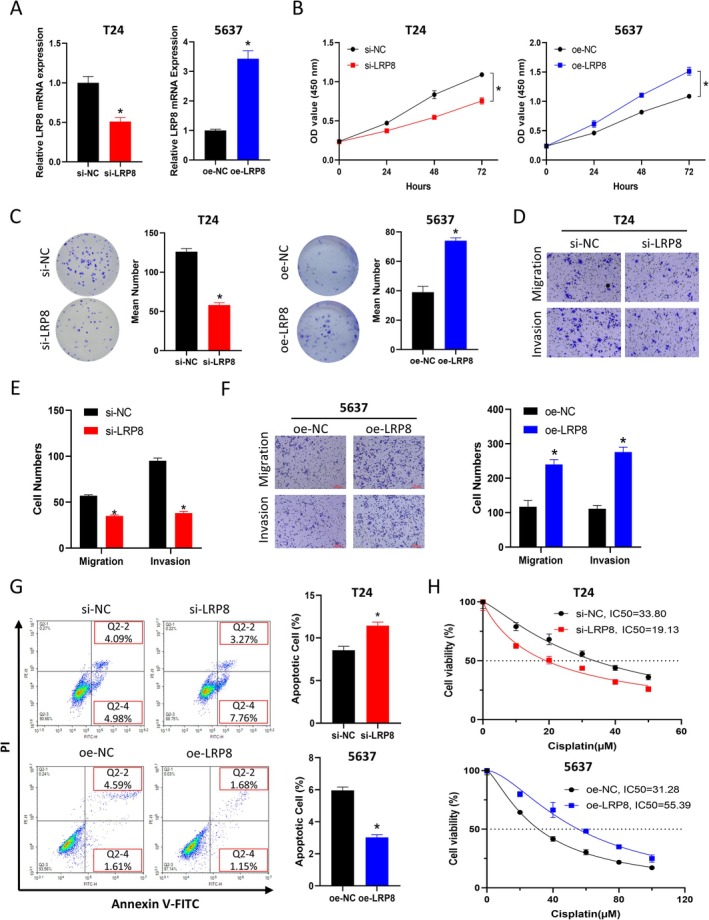
LRP8 promotes malignant progression and cisplatin resistance of BCa cells. (A) QRT‐PCR detection of LRP8 expression. (B) CCK‐8 detection of cell proliferation in each group. (C) Colony formation assay detection of cell proliferation in each group. (D–F) Transwell assay detection of cell migration and invasion ability in each group. (G) Flow cytometry detection of apoptosis ratio in each group. Cells in the Q2‐2 + Q2‐4 area (FITC positive) represent apoptotic cells. (H) IC_50_ analysis of cisplatin resistance sensitivity in each group. * indicates *p* < 0.05, which means a significant difference. All experiments were repeated three times. CCK‐8, Cell Counting Kit‐8; FITC, Fluorescein Isothiocyanate; IC_50_, Half maximal inhibitory concentration; OD, Optical density; PI, Propidium Iodide.

To investigate the role of LRP8 in the malignant progression and chemotherapy resistance of BCa, we constructed a T24 cell line with LRP8 knocked down and a 5637 cell line with LRP8 overexpressed and examined the changes in key indicators. The results of qRT‐PCR suggested that LRP8 was significantly downregulated in the si‐LRP8 group cells and was significantly upregulated in the oe‐LRP8 group (Figure 2A). Then, CCK‐8 and colony formation assays were conducted. The results revealed that the proliferation ability of cells greatly decreased after LRP8 knockdown and was enhanced after LRP8 overexpression (Figure 2B,C). Next, Transwell experiments were performed. The results demonstrated that the ability of cell migration and invasion was significantly weakened after knocking down LRP8 and was significantly enhanced after overexpression of LRP8 (Figure 2D–F). In addition, the apoptosis of cells in each group was assessed in flow cytometry; the proportion of apoptotic cells significantly increased after knocking down LRP8 and significantly decreased after overexpression of LRP8 (Figure 2G). Finally, cells in each group were treated with various concentrations of cisplatin, and the IC50 values were calculated based on cell viability. The cisplatin resistance of tumor cells was repressed after knocking down LRP8 and was enhanced by overexpression of LRP8 (Figure 2H). The above experimental results proved that LRP8 could stimulate the malignant progression and cisplatin resistance of BCa cells.

### 3.3 LRP8 Activates Lipid Metabolism in BCa Cells

**FIGURE 3:**
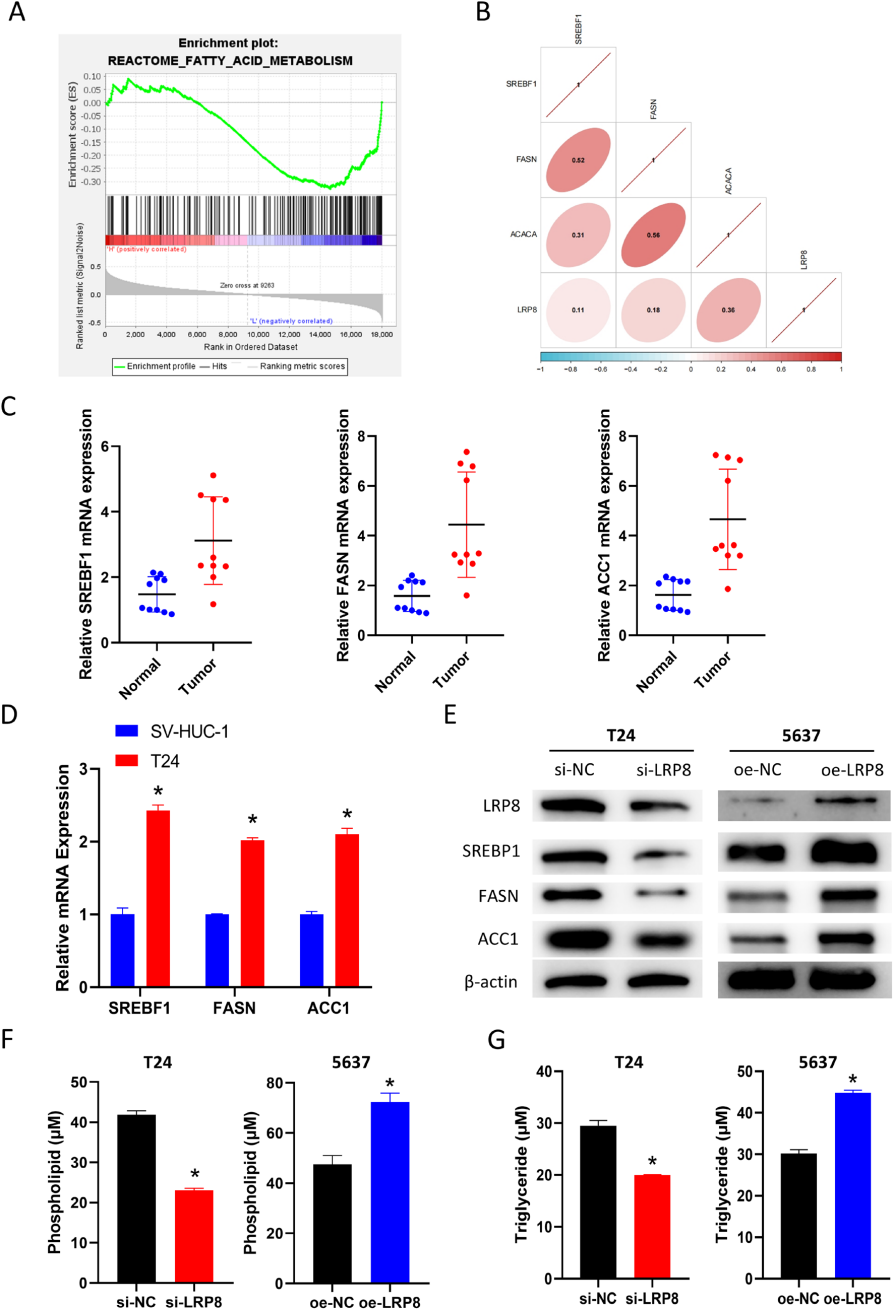
LRP8 regulates lipid metabolism in BCa cells. (A) GSEA pathway enrichment analysis. (B) Pearson correlation analysis. (C) QRT‐PCR detection of lipid metabolism‐related gene in BCa tissues and normal tissues (*n* = 10). (D) qRT‐PCR detection of lipid metabolism‐related gene in BCa cells and normal cells. (E) WB detection of the LRP8 and lipid metabolism‐related protein expression from each group. (F) Phospholipid levels of each group. (G) Triglyceride levels of each group. * indicates *p* < 0.05, which means a significant difference. All experiments were repeated three times. ACC1, Acetyl‐CoA Carboxylase Alpha; FASN, Fatty acid synthase; GSEA, Gene Set Enrichment Analysis; SREBP1, Sterol Regulatory Element‐Binding Protein 1; WB, Western blot.

To investigate the potential mechanisms underlying the role of LRP8, this study utilized GSEA and Pearson correlation analysis to validate the association between LRP8 and lipid metabolism. Furthermore, cellular experiments were conducted to further validate these findings. LRP8 was significantly enriched in the FA metabolism signaling pathway, according to the GSEA results (Figure 3A). Then the correlation between LRP8 and key genes of lipid metabolism (SREBF1, FASN, ACACA/ACC1) was studied. The selection of genes related to lipid metabolism was based on a previous study [16]. A positive correlation between LRP8 and genes involved in lipid metabolism was indicated by Pearson correlation analysis (Figure 3B). Subsequently, this study validated the expression of lipid metabolism‐related genes SREBF1, FASN, and ACC1 in BCa tissues and cells. IHC and qRT‐PCR demonstrated their high expression in BCa tissues and cells (Figure 3C,D). The expression of lipid metabolism‐related proteins (SREBP1, FASN, ACC1) in cells of each group was detected. According to the WB results, lipid metabolism‐related genes were down‐regulated after LRP8 knockdown but were up‐regulated after LRP8 overexpression (Figure 3E). Finally, the levels of phospholipid and triglyceride in cells of each group were examined by the corresponding kits. The results revealed that phospholipid and triglyceride expression levels in cells were significantly reduced after LRP8 knockdown and markedly increased after LRP8 overexpression (Figure 3F,G). These findings indicated that LRP8 could stimulate phospholipid and triglyceride accumulation by activating lipid metabolism in BCa cells.

### 3.4 LRP8 Promotes BCa Malignant Progression and Cisplatin Resistance by Activating Lipid Metabolism

**FIGURE 4:**
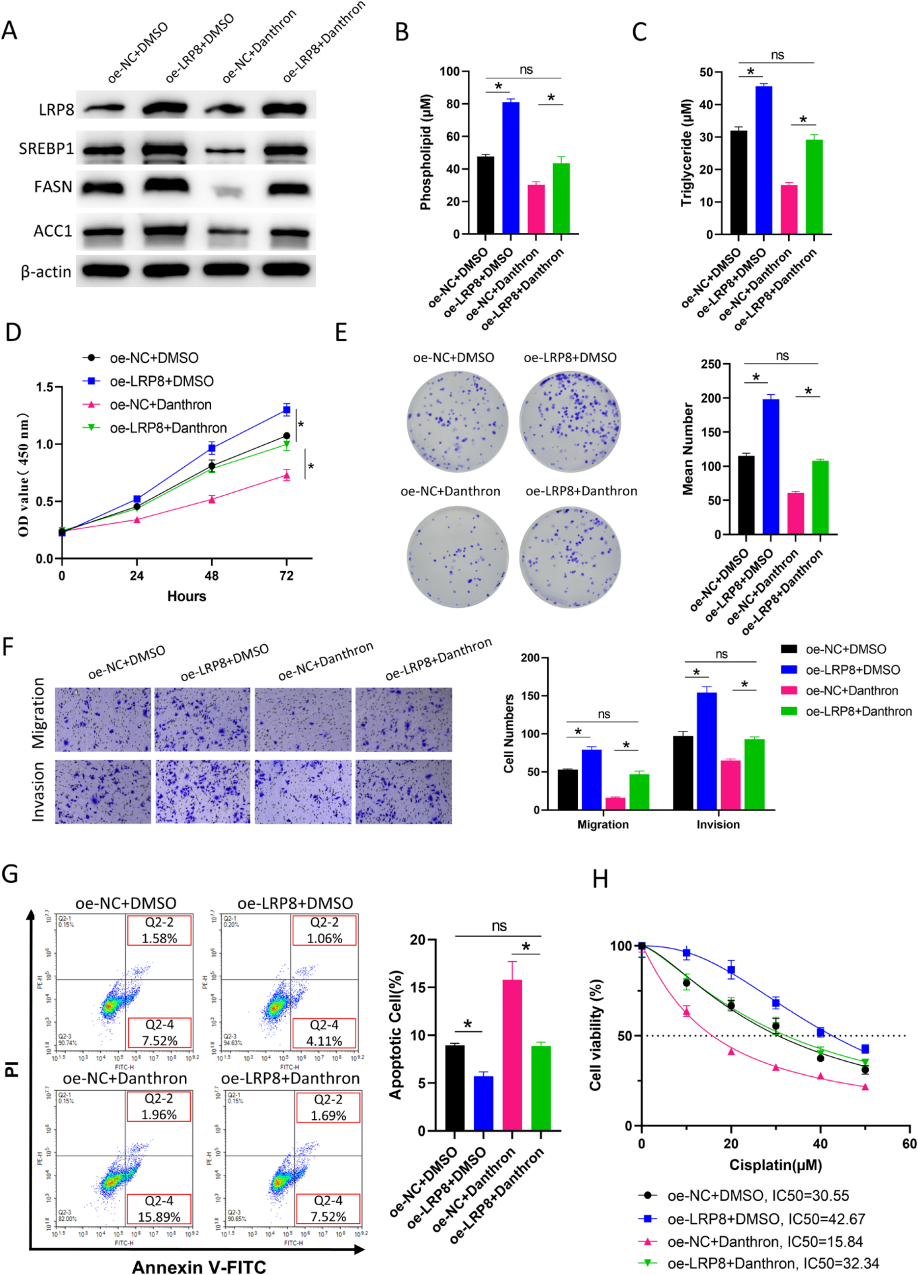
LRP8 promotes BCa malignant progression and cisplatin resistance by regulating lipid metabolism. (A) WB detection of the expression of LRP8 and lipid metabolism‐related proteins in each group. (B) Detection of phospholipid content in each group. (C) Detection of triglyceride content in each group. (D) CCK‐8 detection of cell proliferation in each group. (E) Colony formation assay detection of cell proliferation in each group. (F) Transwell detection of the cell migration and invasion ability in each group. (G) Flow cytometry detection of the cell apoptosis ratio in each group. Cells in the Q2‐2 + Q2‐4 area (FITC positive) represent apoptotic cells. (H) IC_50_ analysis of cisplatin resistance sensitivity in each group. * indicates *p* < 0.05, which means a significant difference; ns indicates no significant difference. All experiments were repeated three times. DMSO, Dimethyl sulfoxide.

To further validate that LRP8 can influence the malignant progression and chemotherapy resistance of BCa through the regulation of lipid metabolism, this study designed rescue experiments and conducted analyses. The 5637 cell line with LRP8 overexpressed combined with lipid metabolism inhibitor (Danthron) was grouped into oe‐NC + DMSO, oe‐LRP8 + DMSO, oe‐NC + Danthron, and oe‐LRP8 + Danthron. The WB results indicated that the expression of lipid metabolism‐related proteins was markedly upregulated by LRP8 overexpression and was downregulated after Danthron treatment. Danthron treatment did not affect the protein expression of LRP8. Lipid metabolism‐related protein expression levels were restored when LRP8 was overexpressed while receiving Danthron treatment (Figure 4A). The lipid content detection results revealed that overexpression of LRP8 markedly raised the phospholipid and triglyceride contents in the cells; however, overexpression of LRP8 with Danthron treatment restored the content of phospholipid and triglyceride to the levels of the control group (Figure 4B,C). The CCK‐8 and colony formation assay results suggested that cell proliferation ability was markedly increased by overexpressing LRP8, but it returned to normal levels when overexpressed LRP8 was treated with Danthron (Figure 4D,E). The Transwell results indicated the migration and invasion ability of cells was greatly increased by overexpressing LRP8, and these abilities were restored when overexpressed LRP8 was simultaneously treated with Danthron (Figure 4F). The results of flow cytometry suggested that cell apoptosis was markedly suppressed by LRP8 overexpression, and the proportion of apoptotic cells returned to normal levels when overexpressed LRP8 was simultaneously treated with Danthron (Figure 4G). Finally, we detected and calculated the IC_50_ values of cisplatin in each cell group. The results indicated that LRP8 overexpression enhanced the cisplatin resistance of cells, while overexpression of LRP8 combined with Danthron treatment restored the cisplatin sensitivity (Figure 4H). The above experimental results evidenced that BCa malignant progression and cisplatin resistance could be stimulated by LRP8 through lipid metabolism activation.

## 4 Discussion

Chemoresistance has always been a major problem during cancer treatment [17], seriously affecting the therapeutic effect of different types of cancer. One of the primary causes of chemoresistance is metabolic reprogramming, among which lipid metabolism reprogramming has received widespread attention [18]. Studies have proved that the synthesis of FAs and cholesterol contributes to maintaining the self‐renewal, differentiation, invasion, and metastasis of tumor stem cells [19, 20], and cancer stem cells are the main drivers of chemotherapy resistance [21]. Furthermore, abnormal expression of various lipid components in drug‐resistant tumor cells can not only regulate the fluidity of the membrane to promote drug binding and efflux but also reduce cell damage caused by oxidative stress [22]. These studies have suggested the key function of lipid metabolism reprogramming in tumor cell chemoresistance. Therefore, regulating lipid metabolism processes could be one potential treatment for BCa chemoresistance. However, safe and efficient regulatory targets have not been found yet.

This study examined the function of LRP8 in the BCa malignant development and chemoresistance to address this problem. The experimental results revealed that the expression of LRP8 was markedly elevated in BCa tissues and cell lines. Knocking down LRP8 dramatically suppressed the malignant progression of BCa cells, while stimulating apoptosis and cisplatin sensitivity in BCa cells. This finding was consistent with relevant reports in other cancers, including ovarian cancer (OC) [23], non‐small cell lung cancer (NSCLC) [24], and pancreatic cancer [25], where the malignant progression of tumor cells is often positively regulated by LRP8. In BC, LRP8 not only activates tumor cell progression [26] but also reduces the proportion of breast stem cells to suppress chemotherapy resistance and metastasis of BC by regulating the Wnt signaling pathway [27].

In addition, overexpression of LRP8 could regulate lipid metabolism in BCa cells, causing phospholipid and triglyceride accumulation, which is often associated with the malignant progression of many cancers, such as GC [28] and OC [29]. Meanwhile, the levels of phospholipid and triglyceride not only predict the development of HCC but also show a negative correlation with the chemotherapy efficacy of sorafenib [30]. These results also confirmed our findings in BCa, that the regulatory role of lipid metabolism affected the BCa malignant progression and cisplatin resistance.

It is worth noting that LRP8 can reinforce the formation of a complex of β‐catenin to mediate the nuclear translocation of β‐catenin, thereby activating the transcription of key genes and regulating the development of GC [31]. This regulatory process is highly likely to be the key to LRP8‐mediated lipid metabolism. The activation of the Wnt/β‐catenin signaling pathway is proven to lead to high expression of lipid metabolism‐related genes [31]. The high expression of lipid metabolism‐related proteins further boosts the activation of the Wnt/β‐catenin signaling pathway. The two form a positive feedback regulation, which facilitates the development and drug resistance of tumors [32, 33]. Of course, the regulatory role of this regulatory mechanism in BCa still needs to be verified by subsequent studies.

In conclusion, this research evidenced that LRP8 was highly expressed in BCa cells and enhanced the malignant progression and cisplatin resistance of BCa cells by regulating lipid metabolism. However, this study still has certain limitations. Firstly, we did not elucidate the specific mechanisms by which LRP8 regulates lipid metabolism. Secondly, validation of LRP8 targets was limited to the cellular level. Therefore, in the next step, we will delve deeper into the relationship between LRP8 and lipid uptake, breakdown, and synthesis, and further validate our conclusions at the animal level. These findings can fill the gap in the field of LRP8 in BCa research while emphasizing that addressing lipid metabolism dysregulation through the supplementation of various nutrients may be a key approach to tackling BCa issues [34].

## Ethics Statement

This study was approved by the Ethics Committee of Shaoxing People's Hospital (17‐01‐2024), approval number: 2024‐Scientific research project 024‐01.

## Conflicts of Interest

The authors declare no conflicts of interest.

## Data Availability

The data that support the findings of this study are available from the corresponding author upon reasonable request.

## References

[1] H. Sung , J. Ferlay , R. L. Siegel , et al., “Global Cancer Statistics 2020: GLOBOCAN Estimates of Incidence and Mortality Worldwide for 36 Cancers in 185 Countries,” CA: A Cancer Journal for Clinicians 71, no. 3 (2021): 209–249. 33538338 10.3322/caac.21660

[2] R. L. Siegel , K. D. Miller , and A. Jemal , “Cancer Statistics, 2018,” CA: A Cancer Journal for Clinicians 68, no. 1 (2018): 7–30. 29313949 10.3322/caac.21442

[3] V. G. Patel , W. K. Oh , and M. D. Galsky , “Treatment of Muscle‐Invasive and Advanced Bladder Cancer in 2020,” CA: A Cancer Journal for Clinicians 70, no. 5 (2020): 404–423. 32767764 10.3322/caac.21631

[4] Z. Cai , F. Zhang , W. Chen , J. Zhang , and H. Li , “miRNAs: A Promising Target in the Chemoresistance of Bladder Cancer,” Oncotargets and Therapy 12 (2019): 11805–11816. 32099386 10.2147/OTT.S231489 PMC6997227

[5] X. Bian , R. Liu , Y. Meng , D. Xing , D. Xu , and Z. Lu , “Lipid Metabolism and Cancer,” Journal of Experimental Medicine 218, no. 1 (2021): e20201606. 33601415 10.1084/jem.20201606 PMC7754673

[6] Y. Cao , “Adipocyte and Lipid Metabolism in Cancer Drug Resistance,” Journal of Clinical Investigation 129, no. 8 (2019): 3006–3017. 31264969 10.1172/JCI127201 PMC6668696

[7] P. Liu , B. Fan , B. Othmane , et al., “M(6)A‐Induced lncDBET Promotes the Malignant Progression of Bladder Cancer Through FABP5‐Mediated Lipid Metabolism,” Theranostics 12, no. 14 (2022): 6291–6307. 36168624 10.7150/thno.71456 PMC9475447

[8] S. Liu , Y. Sun , Y. Hou , et al., “A Novel lncRNA ROPM‐Mediated Lipid Metabolism Governs Breast Cancer Stem Cell Properties,” Journal of Hematology & Oncology 14, no. 1 (2021): 178. 34715882 10.1186/s13045-021-01194-z PMC8555326

[9] M. Y. Lee , A. Yeon , M. Shahid , et al., “Reprogrammed Lipid Metabolism in Bladder Cancer With Cisplatin Resistance,” Oncotarget 9, no. 17 (2018): 13231–13243. 29568353 10.18632/oncotarget.24229 PMC5862574

[10] S. Eissa , M. Swellam , H. el‐Mosallamy , et al., “Diagnostic Value of Urinary Molecular Markers in Bladder Cancer,” Anticancer Research 23, no. 5b (2003): 4347–4355. 14666650

[11] D. Passarella , S. Ciampi , V. Di Liberto , et al., “Low‐Density Lipoprotein Receptor‐Related Protein 8 at the Crossroad Between Cancer and Neurodegeneration,” International Journal of Molecular Sciences 23, no. 16 (2022): 8921. 36012187 10.3390/ijms23168921 PMC9408729

[12] M. D. Waltmann , J. E. Basford , E. S. Konaniah , N. L. Weintraub , and D. Y. Hui , “Apolipoprotein E Receptor‐2 Deficiency Enhances Macrophage Susceptibility to Lipid Accumulation and Cell Death to Augment Atherosclerotic Plaque Progression and Necrosis,” Biochimica et Biophysica Acta 1842, no. 9 (2014): 1395–1405. 24840660 10.1016/j.bbadis.2014.05.009 PMC4125478

[13] J. Cai , J. Chen , T. Wu , et al., “Genome‐Scale CRISPR Activation Screening Identifies a Role of LRP8 in Sorafenib Resistance in Hepatocellular Carcinoma,” Biochemical and Biophysical Research Communications 526, no. 4 (2020): 1170–1176. 32312520 10.1016/j.bbrc.2020.04.040

[14] H. Qiu , X. Shen , B. Chen , et al., “miR‐30b‐5p Inhibits Cancer Progression and Enhances Cisplatin Sensitivity in Lung Cancer Through Targeting LRP8,” Apoptosis 26, no. 5–6 (2021): 261–276. 33779882 10.1007/s10495-021-01665-1

[15] C. Ma , Z. Wang , R. Xia , et al., “Danthron Ameliorates Obesity and MAFLD Through Activating the Interplay Between PPARalpha/RXRalpha Heterodimer and Adiponectin Receptor 2,” Biomedicine & Pharmacotherapy 137 (2021): 111344. 33581653 10.1016/j.biopha.2021.111344

[16] H. Chao , L. Deng , F. Xu , et al., “MEX3C Regulates Lipid Metabolism to Promote Bladder Tumorigenesis Through JNK Pathway,” Oncotargets and Therapy 12 (2019): 3285–3294. 31118679 10.2147/OTT.S199667 PMC6503316

[17] M. M. Mahmoud , E. F. Sanad , and N. M. Hamdy , “Micrornas' Role in the Environment‐Related Non‐Communicable Diseases and Link to Multidrug Resistance, Regulation, or Alteration,” Environmental Science and Pollution Research International 28, no. 28 (2021): 36984–37000. 34046834 10.1007/s11356-021-14550-w

[18] M. Bacci , N. Lorito , A. Smiriglia , and A. Morandi , “Fat and Furious: Lipid Metabolism in Antitumoral Therapy Response and Resistance,” Trends in Cancer 7, no. 3 (2021): 198–213. 33281098 10.1016/j.trecan.2020.10.004

[19] W. Y. Kim , “Therapeutic Targeting of Lipid Synthesis Metabolism for Selective Elimination of Cancer Stem Cells,” Archives of Pharmacal Research 42, no. 1 (2019): 25–39. 30536027 10.1007/s12272-018-1098-z

[20] H. Li , Z. Feng , and M. L. He , “Lipid Metabolism Alteration Contributes to and Maintains the Properties of Cancer Stem Cells,” Theranostics 10, no. 16 (2020): 7053–7069. 32641978 10.7150/thno.41388 PMC7330842

[21] M. Najafi , K. Mortezaee , and J. Majidpoor , “Cancer Stem Cell (CSC) Resistance Drivers,” Life Sciences 234 (2019): 116781. 31430455 10.1016/j.lfs.2019.116781

[22] J. Kopecka , P. Trouillas , A. C. Gasparovic , E. Gazzano , Y. G. Assaraf , and C. Riganti , “Phospholipids and Cholesterol: Inducers of Cancer Multidrug Resistance and Therapeutic Targets,” Drug Resistance Updates 49 (2020): 100670. 31846838 10.1016/j.drup.2019.100670

[23] C. Li , Y. Yang , H. Wang , Y. Song , and H. Huang , “miR‐362‐3p Suppresses Ovarian Cancer by Inhibiting LRP8,” Translational Oncology 15, no. 1 (2022): 101284. 34839107 10.1016/j.tranon.2021.101284 PMC8636862

[24] Z. Fang , M. Zhong , L. Zhou , Y. Le , H. Wang , and Z. Fang , “Low‐Density Lipoprotein Receptor‐Related Protein 8 Facilitates the Proliferation and Invasion of Non‐Small Cell Lung Cancer Cells by Regulating the Wnt/Beta‐Catenin Signaling Pathway,” Bioengineered 13, no. 3 (2022): 6807–6818. 35246020 10.1080/21655979.2022.2036917 PMC8974054

[25] S. Du , H. Wang , J. Cai , et al., “Apolipoprotein E2 Modulates Cell Cycle Function to Promote Proliferation in Pancreatic Cancer Cells via Regulation of the c‐Myc‐p21(Waf1) Signalling Pathway,” Biochemistry and Cell Biology 98, no. 2 (2020): 191–202. 32167787 10.1139/bcb-2018-0230

[26] L. Li , W. H. Qu , H. P. Ma , L. L. Wang , Y. B. Zhang , and Y. Ma , “LRP8, Modulated by miR‐1262, Promotes Tumour Progression and Forecasts the Prognosis of Patients in Breast Cancer,” Archives of Physiology and Biochemistry 128, no. 3 (2022): 657–665. 31994910 10.1080/13813455.2020.1716019

[27] C. C. Lin , M. C. Lo , R. Moody , et al., “Targeting LRP8 Inhibits Breast Cancer Stem Cells in Triple‐Negative Breast Cancer,” Cancer Letters 438 (2018): 165–173. 30227220 10.1016/j.canlet.2018.09.022 PMC6945120

[28] Y. Chi , H. Wang , F. Wang , and M. Ding , “PHTF2 Regulates Lipids Metabolism in Gastric Cancer,” Aging (Albany NY) 12, no. 8 (2020): 6600–6610. 32335542 10.18632/aging.102995 PMC7202541

[29] A. Mukherjee , J. Wu , S. Barbour , and X. Fang , “Lysophosphatidic Acid Activates Lipogenic Pathways and De Novo Lipid Synthesis in Ovarian Cancer Cells,” Journal of Biological Chemistry 287, no. 30 (2012): 24990–25000. 22665482 10.1074/jbc.M112.340083 PMC3408203

[30] W. Dai , L. Xu , X. Yu , et al., “OGDHL Silencing Promotes Hepatocellular Carcinoma by Reprogramming Glutamine Metabolism,” Journal of Hepatology 72, no. 5 (2020): 909–923. 31899205 10.1016/j.jhep.2019.12.015

[31] B. Liu , I. Bukhari , F. Li , et al., “Enhanced LRP8 Expression Induced by *Helicobacter pylori* Drives Gastric Cancer Progression by Facilitating Beta‐Catenin Nuclear Translocation,” Journal of Advanced Research 69 (2025): 299–312. 38609049 10.1016/j.jare.2024.04.002 PMC11954824

[32] J. Wang , R. Ling , Y. Zhou , et al., “SREBP1 Silencing Inhibits the Proliferation and Motility of Human Esophageal Squamous Carcinoma Cells via the Wnt/Beta‐Catenin Signaling Pathway,” Oncology Letters 20, no. 3 (2020): 2855–2869. 32765792 10.3892/ol.2020.11853 PMC7403634

[33] C. O. Kelson , J. W. Tessmann , M. E. Geisen , et al., “Upregulation of Fatty Acid Synthase Increases Activity of Beta‐Catenin and Expression of NOTUM to Enhance Stem‐Like Properties of Colorectal Cancer Cells,” Cells 13, no. 19 (2024): 1663. 39404424 10.3390/cells13191663 PMC11475157

[34] N. M. Hamdy , S. M. Suwailem , and H. O. El‐Mesallamy , “Influence of Vitamin E Supplementation on Endothelial Complications in Type 2 Diabetes Mellitus Patients Who Underwent Coronary Artery Bypass Graft,” Journal of Diabetes and its Complications 23, no. 3 (2009): 167–173. 18413198 10.1016/j.jdiacomp.2007.10.006

